# A randomized controlled trial evaluating low-intensity shockwave therapy for treatment of persistent storage symptoms following transurethral surgery for benign prostatic obstruction

**DOI:** 10.1038/s41391-024-00820-4

**Published:** 2024-03-29

**Authors:** Mohammed Hegazy, Khaled Z. Sheir, Mohamed A. Gaballah, Ahmed M. Elshal

**Affiliations:** Urology and Nephrology Center, Mansoura University, Mansoura, Egypt

**Keywords:** Prostatic diseases, Outcomes research

## Abstract

**Background:**

Low-intensity shockwave therapy (Li-SWT) can improve bladder function through enhancement of angiogenesis and nerve regeneration and suppression of inflammation and overactivity. In this trial, we aimed to evaluate the efficacy of Li-SWT on persistent storage symptoms after transurethral surgery (TUS) for benign prostatic obstruction (BPO).

**Methods:**

Between July 2020 and July 2022, 137 patients with persistent storage symptoms; urgency episodes/24 h ≥ 1 and daytime frequency ≥8, for at least three months after TUS for BPO were randomly allocated to Li-SWT versus sham versus solifenacin 10 mg/day in 3:1:1 ratio. The primary end point was the percent reduction from baseline in overactive bladder symptom score (OABSS) at 3-month follow-up. The changes in 3-day voiding diary parameters, quality of life (QoL) score, peak flow rate and residual urine at 3 and 6-month follow-up were compared. Treatment-related adverse effects were also evaluated.

**Results:**

Baseline data were comparable between groups. The percent reduction from baseline in OABSS at 3-month follow-up was significantly higher in Li-SWT compared to sham (−55% versus −11%), and it was comparable between Li-SWT and solifenacin-10 (−55% versus −60%). Li-SWT achieved significant improvement like solifenacin-10 in 3-day voiding diary parameters and QoL score at 3-month follow-up. This improvement remained comparable between Li-SWT and solifenacin-10 at 6-month follow-up. No adverse effects related to Li-SWT were noted apart from tolerable pain during the procedure. Solifenacin-10 was associated with bothersome adverse effects in 73% of the patients with 11.5% discontinuation rate.

**Conclusions:**

Li-SWT ameliorates persistent storage symptoms and promotes QoL after TUS for BPO, with comparable efficacy and better tolerance compared to solifenacin.

## Introduction

Benign prostatic obstruction (BPO) is a common cause of lower urinary tract symptoms (LUTS) in aging men [1]. LUTS, particularly storage symptoms, interfere with daily activities and negatively impact quality of life (QoL) [2]. After BPO surgery, 20–30% of patients still have persistent storage symptoms [3, 4]. Choi et al. found that persistent storage symptoms after transurethral resection of the prostate was significantly correlated to old age, small bladder capacity and poor detrusor contractility [3]. Mitterberger et al. reported that 30% of patients who presented with preoperative detrusor overactivity (DO) had persistent DO after transurethral resection of the prostate and those patients had reduced bladder perfusion [5]. The pathophysiology of persistent storage symptoms after BPO surgery remains unclear. It may be caused by bladder ischemia and denervation produced by long-standing BPO [5, 6] or it may be related to other factors such as aging [3, 7], chronic inflammation [8] or subtle neurological disorder.

Treatment with muscarinic receptor antagonists (MRAs) or ß3-agonizts is a common practice for storage LUTS after BPO surgery. However, these medications are associated with adverse effects such as dry mouth, constipation and blurred vision after using MRAs or dizziness and blood pressure changes after using ß3-agonizts [9]. After failure of non-invasive treatment, intravesical botulinum toxin-A injection might be considered. However, it may cause urinary tract infection and urine retention, and its effect decreases over time with the need of repeated injection [10].

Low-intensity shockwave therapy (Li-SWT) is a novel non-invasive treatment that has a beneficial effect in improvement of bladder function through induction of angiogenesis, restoration of nerve-ending integrity, suppression of DO and inhibition of inflammatory reactions [11, 12]. Moreover, it has been applied to the penis for erectile dysfunction and to the perineum for chronic prostatitis/chronic pelvic pain syndrome with encouraging results [13, 14]. Recent studies reported that Li-SWT had an important role in improvement of LUTS in patients with benign prostatic hyperplasia [15] and amelioration of overactive bladder symptoms (OABS) in patients with overactive bladder (OAB) [16].

Based on the forementioned studies, it has been proposed that Li-SWT can offer a benefit in improvement of persistent storage symptoms after BPO surgery.

The aim of the current study was to assess the efficacy of Li-SWT for control of persistent storage symptoms after transurethral surgery (TUS) for BPO compared to sham treatment and MRAs (solifenacin).

## Materials and methods

### Study design and enrollment

A randomized controlled trial comparing Li-SWT versus sham versus solifenacin 10 mg was proposed and registered at ClinicalTrials.gov with ID; NCT04437108 (Institutional Review Board approval code: MD.20.06.338). It was conducted in a tertiary care center. Between July 2020 and July 2022, eligible patients were asked to participate in this trial after signing an informed consent form.

Patients with persistent storage symptoms for at least three months following TUS for BPO were screened for inclusion and exclusion criteria. Patients were eligible once they have urgency episodes/24 h ≥ 1 and daytime frequency ≥8, as well as successful relief of bladder outlet obstruction proved mainly by baseline pressure-flow study. Cystometrogram was done to all patients at baseline, however out-patient urethrocystoscopy was performed when pressure-flow study revealed equivocal voiding pattern or when pressure-flow study could not be interpreted due to inability of the patient to void with presence of urodynamic urethral catheter. Patients who had any of the following were excluded: untreated urinary tract infection, neurogenic lower urinary tract dysfunction, uncontrolled diabetes mellitus (Hemoglobin A1c > 6.8), psychogenic disorder, previous pelvic irradiation, prostate or bladder cancer, coagulation disorder, narrow-angle glaucoma, or post-voiding residual urine (PVR) > 150 ml.

### Randomization

Patients were randomly allocated to Li-SWT (group 1) versus sham (group 2) versus solifenacin 10 mg/day (group 3) in 3:1:1 ratio. In group 1, patients were treated by 8-weekly sessions of Li-SWT. This group was divided into three subgroups; suprapubic, perineal and combined, according to the approach through which Li-SWT was applied. In group 2, patients were treated by 8-weekly sessions of sham treatment under conditions like group 1, but with the applicator of the shockwave (SW) device being turned off. Patients in Li-SWT and sham groups were blinded to given treatment. The investigators performed randomization using computer-generated random tables.

### Intervention

Li-SWT was conducted using Dornier AR2 SW device (Dornier MedTech, Wessling, Germany) with a focused SW source. The device was operated by a well-trained urologist. A commercially used gel for sonography was applied to the targeted region.

In suprapubic approach, the bladder was scanned by ultrasound to ensure that it was filled with approximately half of the maximum cystometric capacity measured by baseline cystometrogram, and the patient was asked to lie in flat supine position. The applicator was placed on suprapubic region at three horizontal sites, 2 cm from each other and two fingerbreadths above the pubic bone. In perineal approach, the patient was asked to empty the bladder and lie in lithotomy position. The applicator was placed on perineal region at three vertical sites, 2 cm apart from each other.

Every patient received 3000 shocks/session; 1000 shocks/site in suprapubic or perineal approach, or 1500 suprapubic shocks (500 shocks/site) followed by another 1500 perineal shocks (500 shocks/site) in combined approach, with energy flux density of 0.12 mJ/mm² and frequency of 4 Hz.

### Outcome measures

The primary endpoint was defined as the percent reduction from baseline in the total overactive bladder symptom score (OABSS) at 3-month follow-up; (3-month OABSS minus baseline OABSS)/ baseline OABSS.

The secondary outcome measures included percent of responders in each arm at 3-month follow-up. Three points reduction in the total OABSS was determined as the minimal threshold for a meaningful change [17]. Responders were defined as patients who achieved reduction in the total OABSS ≥ 3 at 3-month follow-up. Other outcome measures included the numerical change from baseline in OABSS (total score and sub-scores), 3-day voiding diary parameters, international prostate symptom score-storage domain (IPSS-S), IPSS-QoL score, maximum flow rate (Qmax) and PVR at 3-month follow-up. Furthermore, changes in OABSS and urodynamic parameters at 6-month follow-up among responders were compared. Also, subgroup analysis was interpreted.

Treatment-related adverse effects were reported. Pain visual analog scale (VAS) ranging from 0–10 was used for pain evaluation during SW sessions.

Treatment failure was considered in patients who failed to achieve reduction in the total OABSS ≥ 3 at 3-month follow-up (non-responders) and patients who discontinued treatment due to intolerable adverse effects. Those patients were offered other treatment options as Li-SWT, solifenacin 10 mg or mirabegron. Nevertheless, this study followed the intention-to-treat analysis.

### Sample size and statistical analysis

The G-power program (University of Dusseldorf, Dusseldorf, Germany) was used to calculate the sample size. Liu et al. showed that solifenacin 5–10 mg/day could achieve >50% reduction from baseline in OABSS at 3-month follow-up in OAB patients [18].

Our hypothesis is that Li-SWT could achieve 50% reduction in OABSS compared to sham. Considering 80% power and α-error probability of 0.05, a sample size of 44 patients (22 patients in each group) was estimated, using a priori test with an effect-size calculation of 0.5 for the x^2^ test. A third group of solifenacin treatment was added as a standard of care (22 patients). Three Li-SWT sub-groups were enrolled as the approach of SW delivery to the bladder is not yet standardized (22 patients in each sub-group). Allowing for 20% drop-out rate, a final number of 135 patients (81: 27: 27) was estimated.

Data analysis was conducted using SPSS V21 (IBM Corp., Armonk, N.Y., USA). Between-group analysis was done using Chi-Square test, fisher’s exact test, one-way ANOVA test, independent sample t-test, Kruskal–wallis H test or Mann–Whitney U test. Within-group analysis was performed using paired sample t-test, Wilcoxon signed-rank test or Chi-Square test. *P* value < 0.05 was considered as the cut-off for statistically significant difference.

## Results

**Fig. 1:**
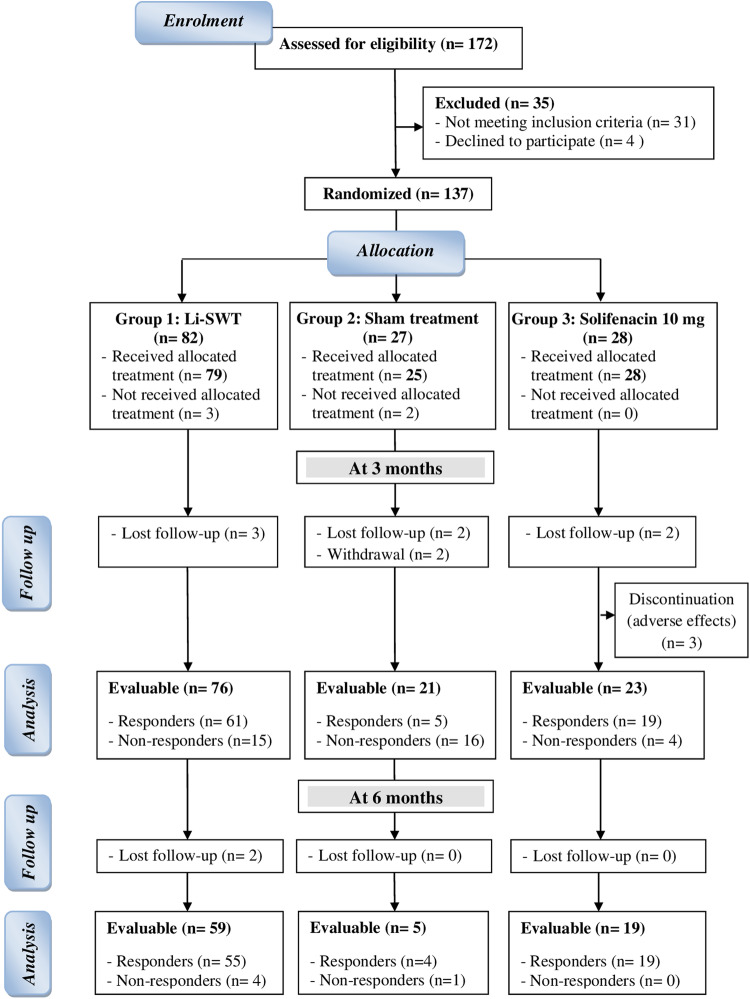
The CONSORT flow chart of the study. It shows evaluable subjects throughout the study phases.

**Fig. 2:**
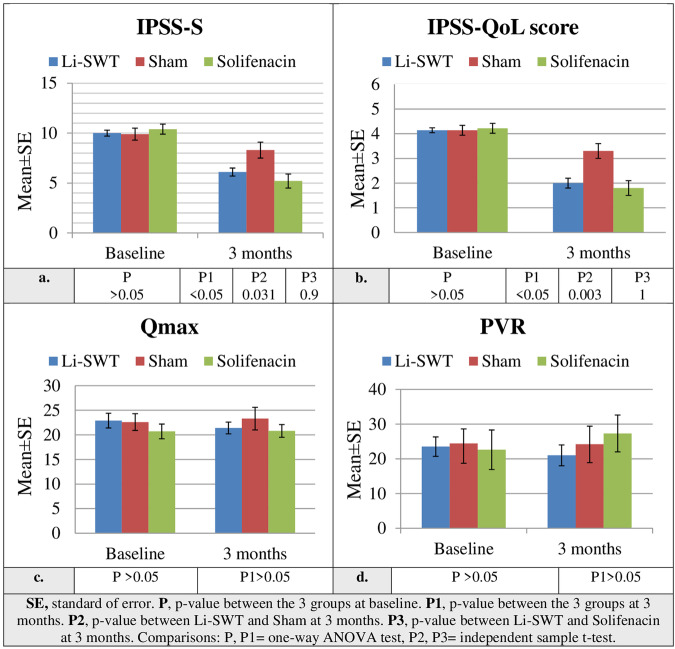
Functional urinary outcome measures among study groups. **a** IPSS; **b** IPSS-QoL score; **c** Qmax; **d** PVR.

Between July 2020 and July 2022, 137 patients were randomly allocated to study groups, as shown in the study’s flow chart (Fig. 1). Patient demographics and peri-operative data of TUS were comparable between study groups (Table 1). There were no significant differences between study groups in the baseline OABSS, 3-day voiding dairy, IPSS-S, QoL score, Qmax, PVR and urodynamic parameters (Tables 2 and 3 and Fig. 2).

**Table 1:** Patients‘ demographics and peri-operative data among study groups.

Variables	Group 1: Li-SWT (*N* = 79)	Group 2: Sham (*N* = 25)	Group 3: Solifenacin 10 mg (*N* = 28)	*P* value
Patients‘ demographics				
Age (mean ± SD) Years	63.4 ± 5.9	64.2 ± 6.3	65.7 ± 4.9	0.195
BMI (mean ± SD) Kg/m^2^	30.6 ± 4.4	28.6 ± 4.9	30.2 ± 4.8	0.169
Diabetes mellitus [*N*(%)]	19 (24.1)	5 (20)	7 (25)	0.896*
Peri-operative data of TUS for BPO				
Indications of TUS [*N*(%)]				
−Refractory LUTS	48 (60.8)	16 (64)	15 (53.6)	0.717*
−Recurrent hematuria	9 (11.4)	3 (12)	3 (10.7)	1.000**
−Retention with failed voiding	20 (25.3)	4 (16)	9 (32.1)	0.397*
−Vesical stone	9 (11.4)	4 (16)	3 (10.7)	0.757**
Interval between start of LUTS and TUS [median (range)] months	28 (6:62)	26 (10:60)	22 (6:65)	0.528
Preoperative PSA [median (range)] ng/dl	2 (0.1:20)	2.3 (0.3: 19)	2.6 (0.4: 23)	0.260
Preoperative prostate biopsy [*N*(%)]	19 (24.1)	7 (28)	5 (17.9)	0.673*
Preoperative prostate size by TRUS [median (range)] ml	54 (21:192)	50 (22:176)	57 (23:156)	0.681
TUS procedure [*N*(%)]				0.578**
−Incision	9 (11.4)	3 (12)	4 (14.3)	
−Resection	48 (60.8)	20 (80)	17 (60.7)	
−Vaporization	3 (3.8)	0 (0)	1 (3.6)	
−Enucleation	19 (24.1)	2 (8)	6 (21.4)	
Early post-operative complications [*N*(%)]				0.551**
−Hematuria managed conservatively	6 (7.6)	1 (4)	3 (10.7)	
−Retention managed by temporarily catheterization	2 (2.5)	1 (4)	1 (3.6)	
−Epididymo-orchitis	0 (0)	1 (4)	0 (0)	
Interval between TUS and study treatment [median (range)] months	7 (3:25)	7 (3:24)	9.5 (3:27)	0.480

Comparisons: one-way ANOVA test for parametric variables, Kruskal–wallis H test for non-parametric variables.

*BMI* body mass index, *TUS* transurethral Surgery, *BPO* benign prostate obstruction, *LUTS* lower urinary tract symptoms, *PSA* prostate specific antigen, *TRUS* transrectal ultrasound.

*P = chi-square test; **P = fisher’s exact test.

**Table 2:** Change from baseline in OABSS and 3-day voiding diary parameters at 3-month follow up.

Variables	Group 1: Li-SWT (*N* = 76)	Group 2: Sham (*N* = 21)	Group 3: Solifenacin (*N* = 23)	*P* value	P1 (G1 vs G2)	P2 (G1 vs G3)
OABSS [median (range)]						
Total score (0–15)						
• Baseline	10 (6–15)	9 (6–13)	10 (7–14)	0.268	–	–
• Change at 3 mo	−5 (−10:0)	−1 (−7:1)	−6 (−10:0)	**0.001**	**0.001**	0.174
Frequency sub-score (0–2)						
• Baseline	1 (1:2)	1 (1:2)	1 (1:2)	0.995	–	–
• Change at 3 mo	−1 (−2:0)	0 (−1:0)	−1 (−1:0)	**0.002**	**0.006**	0.160
Nocturia sub-score (0–3)						
• Baseline	3 (1:3)	3 (1:3)	3 (2:3)	0.202	–	–
• Change at 3 mo	0 (−2:1)	0 (−1:1)	0 (−2:0)	0.151	–	–
Urgency sub-score (0–5)						
• Baseline	4 (3:5)	4 (3:5)	4 (3:5)	0.255	–	–
• Change at 3 mo	−3 (−5:1)	−1 (−4:1)	−3 (−5:0)	**0.001**	**0.001**	0.534
UI sub-score (0-5)						
• Baseline	2 (0:5)	2 (0:4)	2 (0:5)	0.529	–	–
•Change at 3 mo	−1 (−4:1)	0 (−2:2)	−2 (−5:0)	**0.027**	**0.024**	0.343
3-day voiding diary [median (range)]						
Average VV/micturition (ml)						
• Baseline	175 (110:220)	185 (105:215)	180(115:225)	0.804	–	–
• Change at 3 mo	45 (−45:205)	10 (−20:105)	80 (−65:225)	**0.008**	**0.014**	**0.174**
Daytime frequency (times)						
• Baseline	10 (8–18)	9 (8–18)	10 (8–16)	0.897	–	–
• Change at 3 mo	−2 (−12:1)	−1 (−4:3)	−3 (−7:2)	**0.002**	**0.007**	0.107
Nocturia (times)						
• Baseline	4 (1–10)	3 (1–10)	3 (2–11)	0.646	–	–
• Change at 3 mo	−1 (−7:2)	0 (−5:1)	−1 (−8:4)	0.250	–	–
Urgency episodes/24 h						
• Baseline	3 (1–12)	3 (1–10)	3 (1:13)	0.617	–	–
• Change at 3 mo	−2 (−12:1)	0 (−5:2)	−2 (−13:1)	**0.001**	**0.001**	0.355
• UI episodes/24 h	N* = **35**	N* = **9**	N* = **11**			
• Baseline	2 (1:7)	1 (1:3)	2 (1:6)	0.221	–	**–**
• Change at 3 mo	−1 (−7:0)	0 (−2:2)	−1 (−6:1)	**0.006**	**0.002**	0.635

Comparisons: P = Kruskal–wallis H test, P1 & P2 = Mann–Whitney U test.

*OABSS* overactive bladder symptom score, *UI* urgency incontinence, *VV* voided volume, N* number of patients who had baseline UI episodes/24 h ≥1.

Bold values indicates statistical significance.

**Table 3:** Change in urodynamic parameters at 6-month follow up among responders in Li-SWT and solifenacin groups.

Variables	Group 1: Li-SWT group	Group 3: Solifenacin	*P* value**	
Filling cystometry	*N* = 51	*N* = 17		
Capacity at first sensation of bladder filling (mean ± SD) ml				
−Baseline	135 ± 59	144 ± 48		0.579
−6 mo	159 ± 62	163 ± 30		0.802
−*P* value*	**0.002**	**0.031**		
MCC (mean ± SD) ml				
−Baseline	354 ± 108	338 ± 85		0.582
−6 mo	349 ± 102	366 ± 74		0.530
−*P* value*	0.640	0.152		
Compliance (mean ± SD) ml/cmH2O				
−Baseline	35 ± 15	36 ± 15		0.859
−6 mo	38 ± 14	42 ± 16		0.354
−*P* value*	0.075	0.052		
DO [N(%)]				
−Baseline	20 (39.2%)	7 (41.2%)		0.886
−6 mo	10 (19.6%)	5 (29.4%)		0.501
• Still present	8 (15.7%)	5 (29.4%)		
• Newly appeared	2 (3.9%)	0 (0)		
−*P* value*	0.030	0.473		
PFS	*N* = 43	*N* = 15		
Pdet max (mean ± SD) cmH2O				
−Baseline	45 ± 19	45 ± 12		0.956
−6 mo	44 ± 14	44 ± 9		0.970
−*P* value*	0.622	0.761		
BCI (mean ± SD)				
−Baseline	116 ± 37	106 ± 45		0.417
−6 mo	115 ± 34	110 ± 35		0.629
−*P* value*	0.908	0.604		

Comparison: *P (6 mo vs baseline) = paired sample t-test, **P (group 1 vs group 3) = independent sample t-test; this for all variables except DO (chi-square test).

*MCC* maximum cystometric capacity, *DO* detrusor overactivity, *PFS* pressure flow study, *Pdet max* maximum detrusor pressure, *BCI* bladder contractility index.

Bold values indicates statistical significance.

The percent reduction from baseline in the total OABSS at 3-month follow-up (primary end point) was significantly higher in Li-SWT group compared to sham group (−55% versus −11%, *P* = 0.001), and it was not significantly different between Li-SWT and solifenacin groups (−55% versus −60%, *P* = 0.340). The percent of responders at 3-month follow-up was 80.3%, 23.8% and 73.1% in Li-SWT, sham and solifenacin groups respectively (P1; Li-SWT versus sham = 0.001, P2; Li-SWT versus solifenacin = 0.442).

At 3-month follow-up, Li-SWT group achieved better improvement compared to sham group and similar improvement compared to solifenacin group in the change of total OABSS and its frequency, urgency and urgency incontinence sub-scores, as well as average voided volume/micturition, daytime frequency, urgency episodes/24 h, urgency incontinence episodes/24 h, IPSS-S and QoL score. However, there was no significant difference between study groups in nocturia, Qmax and PVR (Table 2 and Fig. 2).

At 6-month follow-up, this efficacy remained comparable between responders in Li-SWT and solifenacin groups. The median percent change from baseline in the total OABSS at 6-month follow-up was −60% in Li-SWT group versus −57% in solifenacin group (*P* = 0.8). Urodynamic analysis revealed significant improvement in cystometric capacity at first sensation of filling among responders in Li-SWT and solifenacin groups (*P* < 0.05), and significant improvement in DO among responders in Li-SWT group (*P* = 0.03) (Table 3).

### Treatment safety

No adverse effects related to Li-SWT were noted apart from local pain during the procedure. The overall VAS (mean ± SD) was 4.22 ± 1.02. Treatment with solifenacin 10 mg was associated with MRAs’ adverse effects in 73% of the patients. Only three patients (11.5%) could not tolerate adverse effects with subsequent discontinuation of treatment. The most common adverse effect was dry mouth (46.2%).

### Subgroup analysis

The numbers of patients treated with Li-SWT through suprapubic, perineal and combined approaches were 27, 25 and 27 patients respectively. The three subgroups had comparable baseline evaluation. At 3-month follow-up, the percent reduction from baseline in the total OABSS (primary end point) was −45, −61 and −53% in suprapubic, perineal and combined subgroups respectively. The difference was significant in all subgroups compared to sham and it was significant favouring perineal against suprapubic subgroup (*P* = 0.020). Otherwise, all other outcome measures were comparable between subgroups. VAS (mean ± SD) was 3.59 ± 0.84, 4.84 ± 1.07 and 4.26 ± 0.76 in suprapubic, perineal and combined subgroups respectively with significantly higher VAS in perineal and combined subgroups compared to suprapubic subgroup (*P* = 0.001 & 0.023 respectively).

## Discussion

After BPO surgery, 20–30% of patients still have persistent storage symptoms [3, 4]. MRAs or ß3-agonizts are the main treatment line, however these medications may result in bothersome adverse effects that might affect patients‘ compliance [9]. Therefore, an alternative and effective treatment option that lacks those adverse effects would be valuable tool. Li-SWT is a non-invasive treatment which will likely improve bladder function through angiogenesis, nerve regeneration and suppression of inflammation [11, 12]. There are some clinical studies that used Li-SWT to treat LUTS [19]. However, no study has been conducted using Li-SWT to treat post prostatectomy storage symptoms.

In 2019, Zhang et al. used radial extracorporeal SW therapy through perineal approach to treat patients with benign prostatic hyperplasia [15]. The patients received 2000 shocks, once/week for 8 weeks, at two bar and frequency of 10 Hz. The study revealed significant improvement compared to baseline in IPSS and QoL score at 4 weeks and became sustained through the 3-month follow-up.

In 2021, Lu et al. compared the effect of 8-weekly sessions of Li-SWT versus sham on females with OAB [16]. Focused SWs were applied through suprapubic approach using 3000 shocks/session, energy flux density of 0.25 mJ/mm^2^ and frequency of 3 Hz. The authors found that Li-SWT achieved significant improvement in OABSS, daytime frequency and QoL questionnaire at 4 weeks compared to sham and significant improvement in average voided volume/micturition, functional bladder capacity and all OABS at 8 weeks. This improvement remained constant till 6 months after treatment.

Herein, Li-SWT was associated with significant improvement in OABSS, daytime frequency, urgency, urgency incontinence, average voided volume/micturition and QoL score compared to sham. The three approaches of Li-SWT had similar efficacy apart from significantly higher percent reduction in OABSS at 3-month follow-up in perineal compared to suprapubic approach.

Solifenacin improves persistent storage symptoms after prostatectomy [20, 21]. In the present study, solifenacin 10 mg improved all OABS apart from nocturia, with comparable efficacy to Li-SWT. The dose of 10 mg was used because all included patients had tried initially different treatment regimens including solifenacin 5 mg. Two weeks washout period was offered to all patients before randomization.

Unlike previous studies [16, 22], there was no significant improvement in nocturia in Li-SWT and solifenacin groups in the current study. However, Iselin et al. similarly showed that the use of oxybutynin early after transurethral resection of the prostate improved storage symptoms except for nocturia [23]. This might be secondary to the difference in study population and pathophysiology of nocturia [24].

In the current study, the change in OABSS was compared using both actual and percent reduction from baseline, and the significance was similar in both. The primary end point was considered as the percent reduction in OABSS at 3-months resembling the study of Liu et al. [18] that used it to express the effect of solifenacin in OAB patients.

Unlike data of Zhang et al. [15] and Lu et al. [16], the current study revealed that Li-SWT had no effect on Qmax and PVR. Nevertheless, the study of Zhang et al. was a non-randomized trial including patients with benign prostatic hyperplasia and the effect on Qmax and PVR was assessed at 4 and 8 weeks only. Along with the current study, a recent meta-analysis found that Li-SWT does not improve Qmax and PVR compared to sham in patients with chronic pelvic pain syndrome [19]. Also, solifenacin does not affect Qmax and PVR in OAB patients [25], and similar results were found in the present study.

Considering urodynamic changes, the impact of Li-SWT and solifenacin on bladder function was assessed among responders at 6-months. Both treatment modalities could increase cystometric capacity at first sensation of filling. Li-SWT resulted in significant decrease in the percent of patients who had DO compared to baseline (19.6% versus 39.2%).

The safety of Li-SWT was documented by previous studies [15, 16, 19] and confirmed by the present study. Zhang et al*.* reported slight perineal pain after perineal application of radial extracorporeal SW therapy [15]. Also, the pain score in the present study was significantly higher in perineal and combined approaches than suprapubic approach. This may be due to compression of the tissues against pelvic bones. Chapple et al*.* reported 27.7% dry mouth and 6.8% discontinuation rate with solifenacin 10 mg [22], and it was 46.2% and 11.5% respectively in the current study.

Up to date, there is no ideal treatment protocol for Li-SWT regarding application site, energy flux density, number of shocks and sessions and time interval between sessions. Also, there were no reported difference among SW energy generators and handles [26]. Following this trial, the combined approach including all the focal areas through perineal and suprapubic approaches was protocoled in our institute. This could maximize efficacy and reduce pain. Patients with post prostatectomy persistent storage symptoms will be offered Li-SWT whenever they fail or experience adverse effects with bladder targeting medicines.

Study’s limitations include the short-term follow-up. Moreover, proper assessment of nocturia was not performed and QoL was evaluated by a single question rather than specific QoL questionnaires. Lack of evaluation of the cost difference between the two treatment modalities is another limitation.

Nevertheless, the present study is the first trial evaluating the efficacy of Li-SWT on persistent storage symptoms after BPO surgery. Trying different approaches of Li-SWT provided valuable information and the use of focused SWs was helpful to avoid dispersion of SWs [27]. Herein, the use of urodynamic evaluation objectively confirmed the theory. Therefore, this study serves as a motive for further clinical trials examining the need for more SW sessions on long term. Also, the use of color doppler ultrasound might be useful in future studies to define the impact of Li-SWT on bladder vascularity.

In conclusion, Li-SWT is effective and safe treatment method for persistent storage symptoms after TUS for BPO. It ameliorates OABS including frequency, urgency and urgency incontinence and improves QoL. It can be used as an alternative option to MRAs with similar efficacy and less bothersome adverse effects.

## Data Availability

The datasets generated during and/or analyzed during the current study are available from the corresponding author on reasonable request.
